# Polymorphic insertions of *DcSto* miniature inverted-repeat transposable elements reveal genetic diversity structure within the cultivated carrot

**DOI:** 10.1007/s13353-024-00916-6

**Published:** 2024-10-28

**Authors:** Santosh Hadagali, Katarzyna Stelmach-Wityk, Alicja Macko-Podgórni, Sarvamangala Cholin, Dariusz Grzebelus

**Affiliations:** 1 Department of Plant Biology and Biotechnology, University of Agriculture in Krakow, Al. Mickiewicza 21, 31-120 Kraków, Kraków, Poland; 2 Plant Molecular Biology Lab (DBT-BIOCARe), Dept. of Biotechnology and Crop Improvement, College of Horticulture, University of Horticultural Sciences, Bagalkot, Karnataka 587103 India

**Keywords:** *Daucus carota*, Diversity, Intron length polymorphism, MITE, Mobile DNA

## Abstract

Miniature inverted-repeat transposable elements (MITEs) are a potent source of polymorphisms in plant genomes. A genotyping system, named *DcS*-ILP, based on polymorphic insertions of *Stowaway* MITEs (*DcSto*s) localized in introns and identified in the reference genome DH1, has been developed for carrot. Here, we report an extension of the *DcS*-ILP genotyping system by incorporation of non-reference insertions identified in resequenced genomes representing the eastern gene pool. We genotyped 52 carrot accessions representing the eastern and western carrot gene pools with 92 markers developed previously (western *DcS*-ILP panel) together with 84 newly developed markers (eastern *DcS*-ILP panel). Overall, the *DcS*-ILP markers revealed a highly structured genetic diversity separating the eastern and the western carrot accessions at *K* = 2 and differentiating Indian breeding lines from the eastern accessions at *K* = 3. The eastern *DcS*-ILP panel proved to be more robust with respect to the eastern carrot gene pool, while it provided little information on the western accessions, as many of the *DcSto* insertions present in the eastern gene pool were absent in the western gene pool. As the western carrot accessions represent improved cultivars, *DcSto* insertional polymorphisms allowed detection of a selection-driven bottleneck at the improvement stage. Selection in the course of the improvement stage generally operated on standing variation, as the subset of *DcSto* insertions present in the western carrot likely originated from transposition events preceding the separation of both gene pools. However, occasional frequency shifts in the opposite direction were also revealed, possibly indicating selection for favorable variants associated with *DcSto* insertions.

**Supplementary information:**

The online version contains supplementary material available at 10.1007/s13353-024-00916-6.

## Introduction

Carrot is in top ten of the most economically important vegetables worldwide in terms of the area of production and market value. Its production and consumption, either raw or processed, have been continuously growing in recent decades (Simon 2019). Orange-colored carrots are a primary source of β-carotene in the western diet and a rich source of carbohydrates, proteins, vitamins, and minerals.

Carrot (*Daucus carota* L.) belongs to the order Apiales and the family Apiaceae. It is a diploid species with chromosome number 2*n* = 2*x* = 18 and a relatively small genome of 473 Mb (Coe et al. 2023). It is an outcrossing species showing high inbreeding depression. Several carrot subspecies have been recognized (Spooner 2019) including the cultivated carrot classified as *D. carota* subsp. *sativus*. Carrot was domesticated approximately 1100 years ago in western or central Asia (Iorizzo et al. 2013). Early domesticates, nowadays represented by Asian landraces, referred to as eastern carrots, are usually yellow- or purple-rooted, as they accumulate lutein and/or anthocyanins, respectively. In contrast, improved cultivars, referred to as western carrots and grown in Europe and Americas, are usually orange-rooted due to the presence of carotenes. In comparison to their eastern counterparts, they are characterized by more uniform, cylindrical or tapered cylindrical roots, lower tendency to root branching, and higher vernalization requirements (Rubatzky et al. 1999). Most likely, orange-rooted cultivars first appeared in Europe only in the seventeenth century; hence, carotene accumulation was revealed to be a relatively recent improvement. More recently, breeding efforts have focused on broadening of the carrot cultivation area by developing cultivars of western type better adapted to production in sub-tropical regions of South America, e.g., “Brasilia” in Brazil or “Criolla” in Argentina. Similar breeding programs are currently being carried out in India.

Transposable elements (TEs) are entities capable of changing their location in the host genome (Piégu et al. 2015). Despite being recognized as selfish elements, TEs were shown to play important roles in adaptation to new environmental conditions, providing plasticity to plant genomes and crucial innovations to crops (Jangam et al. 2017; Kidwell & Lisch 1997). For example, TE insertions produced gene knock-outs resulting in recessive mutations required for the accumulation of carotenoids in carrot roots (Hadagali and Grzebelus 2024).

The TEs are divided into two classes: class-I elements (retrotransposons) are mobilized via a “copy and paste” mechanism utilizing an RNA intermediate, while class-II elements (DNA transposons) transpose by physical excision and reintegration. In carrot, roughly a half of the genome is occupied by TEs, most of which are long terminal repeat (LTR) retrotransposons. Miniature inverted-repeat transposable elements (MITEs) are small (usually less than 600-bp long) non-autonomous elements grouped into families, sometimes comprising thousands of copies. They are derivatives of DNA transposons flanked by terminal inverted repeats (TIRs). As such, MITEs can be mobilized by their autonomous relatives. The carrot genome is rich in MITEs both in terms of their diversity and copy numbers, comprising 428 MITE families and more than 31 thousand copies, collectively spanning 10.34 Mb (Macko-Podgórni et al. 2021). Among those, a group of *DcSto* (*D. carota Stowaway*-like) MITEs has been investigated in more detail and they were shown to be frequently positioned in a vicinity of genes and highly polymorphic (Macko-Podgórni et al. 2019). Thus, we developed a genotyping panel based on insertional polymorphisms of *DcSto* MITEs residing in introns, named *DcS*-ILPs (*Daucus carota Stowaway*-like intron length polymorphisms; Stelmach et al. 2017) and successfully applied them to elucidate the genetic structure of western carrots representing different market types (Stelmach et al. 2021).

As the original *DcS*-ILP panel comprising more than 100 polymorphisms was designed only with the use of insertions present in the reference genome DH1—a doubled haploid line developed from a western carrot of the Nantes market type (Iorizzo et al. 2016), we considered it highly biased towards the western carrot group. Thus, in the present investigation, we developed an additional panel of *DcS*-ILPs based on insertional polymorphisms of *DcSto* copies present in resequenced genomes of eastern carrots but absent in the reference genome, as revealed by Macko-Podgórni et al. (2019). Finally, we compared the performance of both *DcS*-ILP genotyping panels with respect to their ability to elucidate genetic diversity and population structure of a collection of eastern and western accessions.

## Material and methods

### Plant materials

The study included a total of 52 accessions. Among those, 32 accessions represented the eastern gene pool, including 10 Indian breeding lines originating from a breeding program carried out at the University of Horticultural Sciences in Bagalkot. The remaining 20 accessions represented the western carrot gene pool. One plant per accession was genotyped (Table 1). Seeds were sown in pots in the greenhouse. Young leaves were collected, and DNA was extracted from fresh samples using a modified CTAB protocol (Briard et al. 2000). DNA concentration was estimated using NanoDrop 2000 (Thermo Fisher Scientific). DNAs were diluted to a working concentration of 50 ng/μl and stored at − 20 °C.

**Table 1:** A list of carrot accessions used in the study

Code	Gene bank ID	Gene pool	Cv. name or status	Source*	Country of origin
E01	3931	Eastern	Persia No 242	HRIGRU	Iran
E02	4009	Eastern	Annual Red Rawalpindi	HRIGRU	Pakistan
E03	6682	Eastern	Landrace	HRIGRU	Azerbaijan
E04	6684	Eastern	Landrace	HRIGRU	Former USSR
E05	7301	Eastern	Landrace	HRIGRU	India
E06	10156	Eastern	Landrace	HRIGRU	India
E07	10190	Eastern	Landrace	HRIGRU	Turkey
E08	10217	Eastern	Landrace	HRIGRU	Turkey
E09	10220	Eastern	Landrace	HRIGRU	Syria
E10	10228	Eastern	Landrace	HRIGRU	India
E11	10257	Eastern	Landrace	HRIGRU	Afghanistan
E12	10,260	Eastern	Landrace	HRIGRU	Afghanistan
E13	10264	Eastern	Landrace	HRIGRU	Pakistan
E14	10273	Eastern	Landrace	HRIGRU	Iran
E15	10275	Eastern	Landrace	HRIGRU	Iran
E16	10308	Eastern	Landrace	HRIGRU	Afghanistan
E17	10331	Eastern	Kintoki	HRIGRU	Japan
E18	10522	Eastern	Landrace	HRIGRU	China
E19	10524	Eastern	Landrace	HRIGRU	China
E20	12601	Eastern	T-29	HRIGRU	Pakistan
E21	13204	Eastern	Landrace	HRIGRU	Former USSR
E22	13208	Eastern	Wild	HRIGRU	Former USSR
EI01	IC-0646404	Eastern	Breeding line	UHSB	India
EI02	IC-0646405	Eastern	Breeding line	UHSB	India
EI03	IC-0646406	Eastern	Breeding line	UHSB	India
EI04	IC-0646407	Eastern	Breeding line	UHSB	India
EI05	IC-0646408	Eastern	Breeding line	UHSB	India
EI06	IC-0646409	Eastern	Breeding line	UHSB	India
EI07	IC-0646410	Eastern	Breeding line	UHSB	India
EI08	IC-0646411	Eastern	Breeding line	UHSB	India
EI09	IC-0646412	Eastern	Breeding line	UHSB	India
EI10	IC-064613	Eastern	Breeding line	UHSB	India
W01	7123	Western	Foram	HRIGRU	The Netherlands
W02	3942	Western	Amsterdam Grace	HRIGRU	Denmark
W03	5477	Western	Amsterdam Forcing	HRIGRU	Great Britain
W04	6030	Western	Pickmo	HRIGRU	Sweden
W05	3970	Western	Amstel	HRIGRU	France
W06	8860	Western	Chantenay Royal	HRIGRU	France
W07	3882	Western	Royal Chantenay	HRIGRU	USA
W08	4004	Western	Shin Kuroda Gosun	HRIGRU	Japan
W09	4670	Western	Macbeth	HRIGRU	Great Britain
W10	7255	Western	Criolla	HRIGRU	Argentina
W11	7131	Western	Davanture	HRIGRU	France
W12	5596	Western	Paris Market	HRIGRU	The Netherlands
W13	6490	Western	Early Scarlet Horn	HRIGRU	Great Britain
W14	9294	Western	Parijse Markt	HRIGRU	The Netherlands
W15	9296	Western	Parijse Markt (Rubin)	HRIGRU	The Netherlands
W16	3982	Western	Red Elephant	HRIGRU	Great Britain
W17	6514	Western	St. Valery	HRIGRU	Great Britain
W18	6788	Western	St. Valery	HRIGRU	Poland
W19	6004	Western	Kieler Rote	HRIGRU	Germany
W20	6163	Western	New Red Intermediate	HRIGRU	Great Britain

*HRIGRU, Warwick Genetic Resources Unit, Wellesbourne, Warwick, United Kingdom; *UHS*, University of Horticultural Sciences, Bagalkot, Karnataka, India

### Development of DcS-ILP markers from non-reference DcSto insertions

Eleven resequenced genomes comprising five wild accessions and six landraces representing the eastern carrot gene pool (Iorizzo et al. 2016) were used to search for intronic insertions of *DcSto* elements. Genomic coordinates of *DcSto* insertions identified in at least one of the resequenced genomes and absent in the reference genome were retrieved from the data reported by Macko-Podgórni et al. (2019) and compared to coordinates of genes annotated in the carrot reference DH1 genome assembly (Iorizzo et al. 2016; NCBI acc. no. LNRQ01000000). Four hundred and nine gene-associated intron-localized *DcSto* insertion sites were identified, of which 169 were manually selected for the development of *DcS*-ILP markers. The criteria for marker selection were as follows: insertion sites were (1) free from any other annotated repetitive sequences, (2) evenly distributed over each chromosome, and (3) present in introns less than 2.8-Kb long. Primer-BLAST (Ye et al. 2012) was used to design PCR primer pairs anchored in exons flanking the introns of interest. Primer pairs were designed to amplify fragments ranging from 400 to 2900 bp. The optimal annealing temperature was set to 58 °C. Validation of candidate *DcS*-ILP markers was performed on eight accessions representing the eastern gene pool, as described by Stelmach et al. (2017). Eighty-four positively validated markers, i.e., those producing clear length polymorphisms were used in subsequent stages.

### DcS-ILP marker genotyping

The total of 176 *DcS*-ILP markers was used in the present study, including 92 markers developed previously by Stelmach et al. (2017) based on *DcSto* insertions present in the carrot reference genome DH1, hereafter referred to as the “western *DcS*-ILP panel” (Supplementary Table S1), and 84 markers newly developed from non-reference insertions found in 11 resequenced genomes of eastern type carrots (Macko-Podgórni et al. 2019), hereafter referred to as the “eastern *DcS*-ILP panel” (Supplementary Table S2). Eppendorf MasterCycler Gradient was used for the PCR amplification with the following thermal profile: 94 ℃ (120 s), 30 cycles of 94 ℃ (30 s), 56 ℃ (30 s), 68 ℃ (120 s), and final step of 68 ℃ (600 s). The total volume of each PCR product was 10 μl. The PCR components consisted of 50 ng genomic DNA, 0.5 μM of forward and 0.5 μM reverse primer, 0.5 U Taq DNA polymerase (Thermo Fisher Scientific), 0.25 mM of each dNTP (Thermo Fisher Scientific), and 1 × Taq buffer. Annealing temperatures were adjusted for each primer pair, as shown in Supplementary Tables S1 and S2. Agarose gel electrophoresis was used to separate PCR products (1% gel, 1xTBE buffer, separation for 2 h at 120 V). The DNA was visualized using SYBR gel stain (Thermo Fisher Scientific) under UV, and electrophoregrams were documented as digital images. Polymorphisms were scored manually as described by (Stelmach et al. 2017). All polymorphisms were bi-allelic, as they reflected the presence/absence of *DcSto* copies; hence, the longer PCR product was scored as the “occupied” variant and encoded “1,” while the shorter product represented the “empty” variant and was encoded as “0” (Supplementary Tables S3-S6).

### Evaluation of DcS-ILP markers performance and inference of the carrot genetic diversity structure

Number of alleles (Na), number of effective alleles (Ne), information index (I), observed and expected heterozygosity (Ho and He), percentage of polymorphic loci (%P), and fixation index (F) were estimated using GenAlEx 6.51 (Peakall and Smouse 2012).

STRUCTURE 2.2.3 (Pritchard et al. 2000) was used to infer the genetic diversity structure. No prior information on the origin of accessions was used. The following parameters were set for analysis: 10^5^ iterations for burn-in and 10^5^ iterations for Markov Chain Monte Carlo (MCMC). The assumed number of populations (K) was set from 1 to 10, and the admixture model with correlated allelic frequencies among populations was applied. The most probable *K* value was determined using Structure Harvester (Earl and von Holdt 2012). This approach utilized the Δ*K* method, which is an ad hoc statistic based on the rate of change in the log probability of data across different cluster numbers, as described by Evanno et al. (2005). Distruct 1.1 (Rosenberg 2003) was used to display STRUCTURE results. Principal coordinate analysis (PCoA) was conducted in GenAlEx 6.51 (Peakall and Smouse 2012).

## Results

### Development and validation of the eastern DcS-ILP panel

**Fig. 1:**
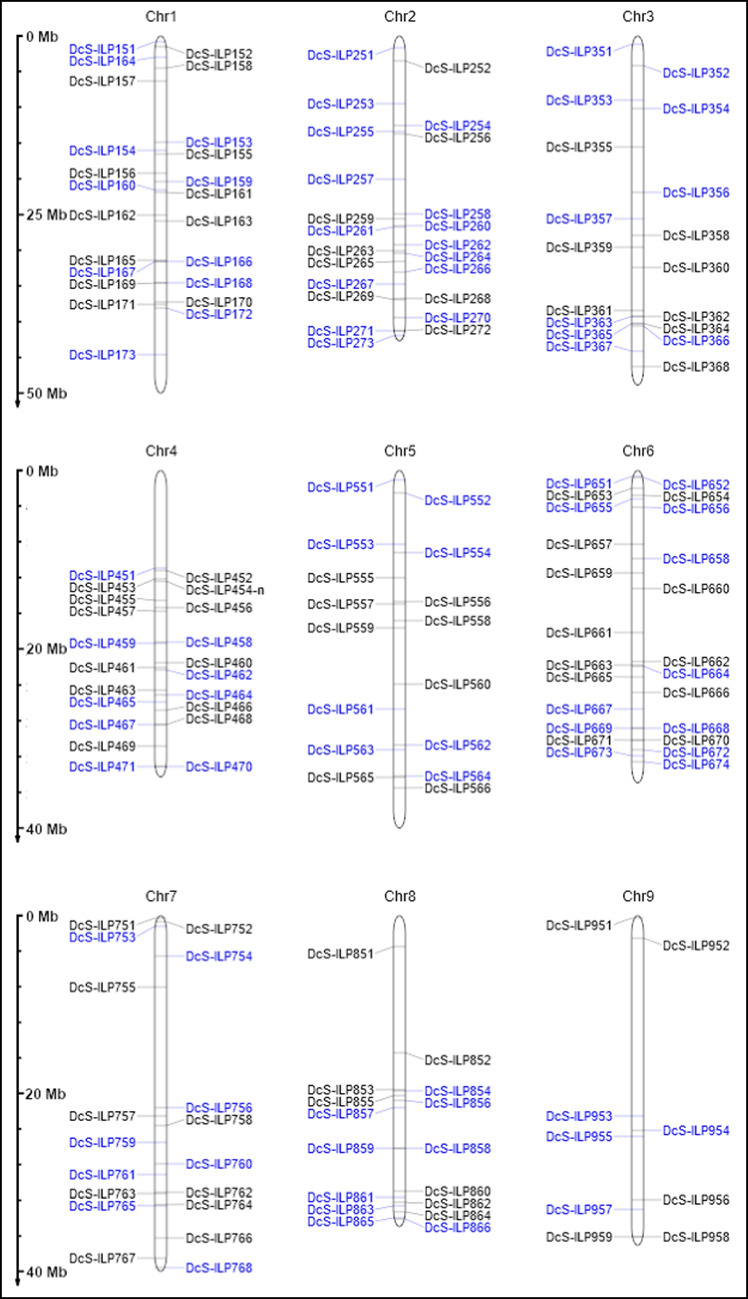
Distribution of 169 *DcSto* copies used to develop the eastern *DcS*-ILP panel on nine carrot chromosomes. The 84 polymorphic *DcSto* insertions used for genotyping (constituting the “eastern *DcS*-ILP panel”) are highlighted in blue

Coordinates of 409 *DcSto* copies present in any of the 11 resequenced genomes representing the eastern carrot gene pool but absent in the DH1 reference genome were compared with coordinates of all genes annotated in the DH1 genome assembly (Iorizzo et al. 2016). Of those, 169 matched the *DcS*-ILP marker development criteria specified in the methods section. Following an initial screening using eight accessions of eastern carrot, 84 *DcSto* insertions were confirmed to be polymorphic and were used to genotype the whole collection. The 84 markers were distributed on all carrot chromosomes, ranging from 4 on chromosome 9 to 15 on chromosome 2 (Fig. 1).

### Inference of the genetic diversity structure in the cultivated carrot

**Fig. 2:**
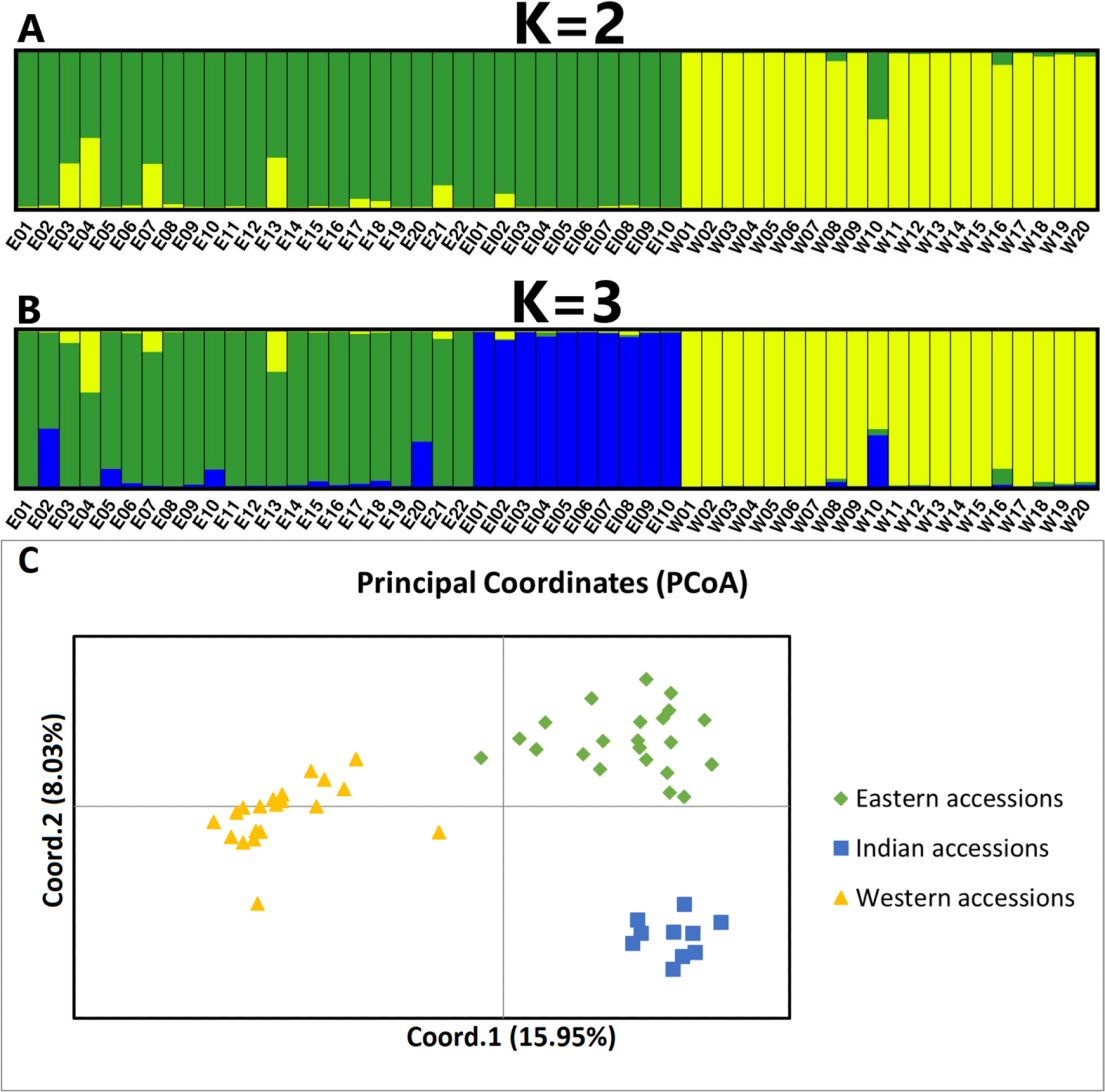
Genetic diversity structure in a collection of 52 carrot accessions representing eastern (E01-E22), Indian (EI01-EI10), and western (W01-W20) gene pools using the combined information from western and eastern *DcS*-ILP panels (the total of 176 markers). STRUCTURE results at *K* = 2 (**a**) and *K* = 3 (**b**) and PCoA results (**c**) are shown

The genetic diversity structure revealed by the combination of the western and eastern *DcS*-ILP genotyping panels, i.e., based on the total of 176 *DcSto* insertional polymorphisms, clearly separated the western carrot gene pool from the eastern gene pool at *K* = 2 (Fig. 2a), while at *K* = 3, the breeding lines of the Indian origin were grouped into a well-separated cluster (Fig. 2b). Very little admixture was observed in the western carrot gene pool, except cv. Criolla (W10), an Argentinian cultivar adapted to cultivation in the subtropical climate. Apparently, the admixture could be attributed to the use of plant materials with relaxed vernalization requirements in the breeding history of that cultivar. In the eastern carrot gene pool, landraces originating from the former USSR (E04) and Pakistan (E13) indicated a degree of admixture. PCoA analysis also showed non-overlapping clusters of accessions representing western and eastern carrot gene pools and a separate cluster grouping the lines from the Indian breeding program (Fig. 2c).

**Fig. 3:**
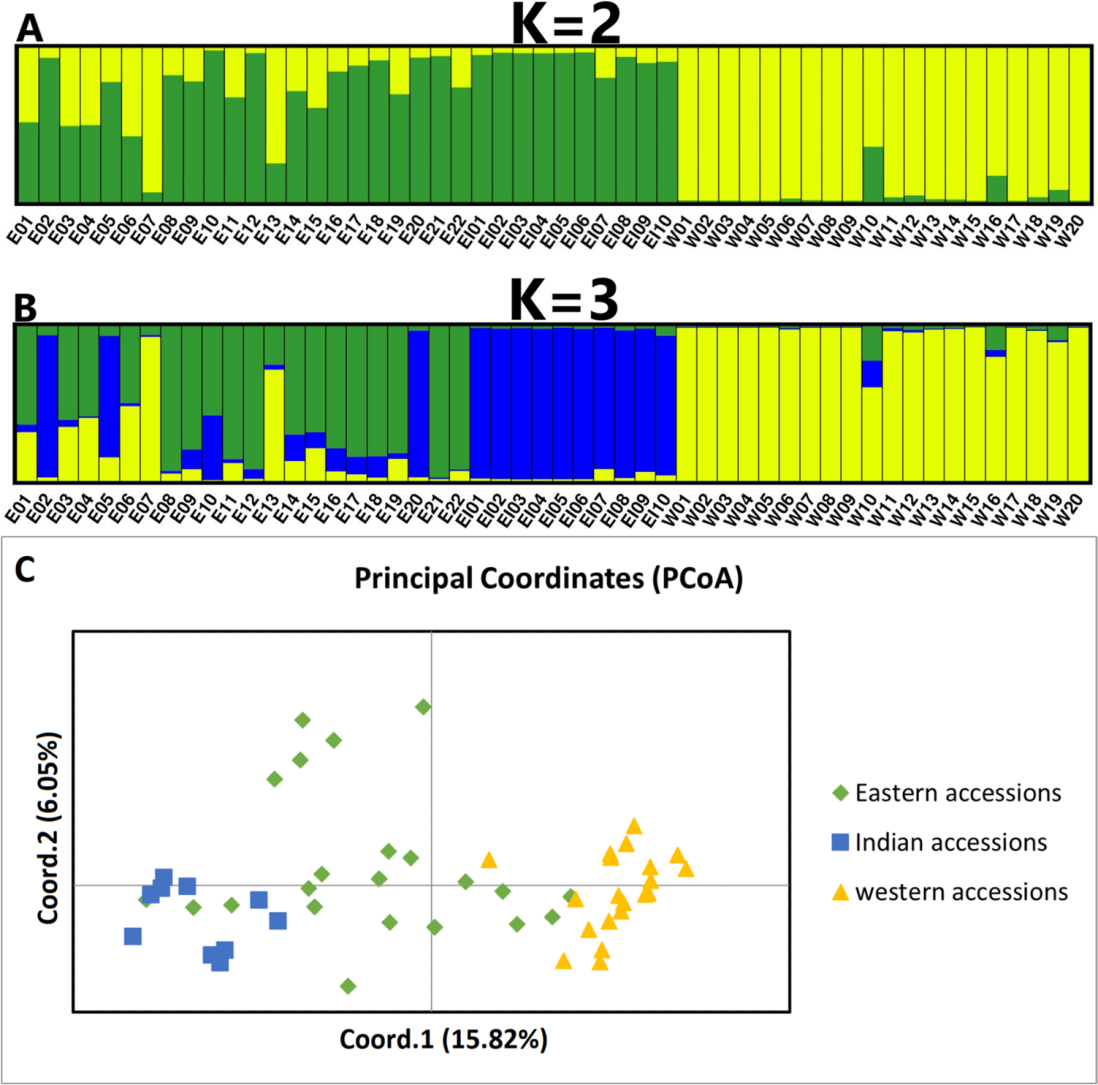
Genetic diversity structure in a collection of 52 carrot accessions representing eastern (E01-E22), Indian (EI01-EI10), and western (W01-W20) gene pools genotyped with 84 markers from the eastern *DcS*-ILP panel. STRUCTURE results at *K* = 2 ( **a**) and *K* = 3 (**b**) and PCoA results (**c**)) are shown

The use of 84 non-reference polymorphic *DcSto* insertions, i.e., those absent in the DH1 genome and constituting the eastern *DcS*-ILP genotyping panel (Supplementary Tables S3-S4), revealed a more complex structure of genetic diversity in the eastern carrot gene pool. At *K* = 2, separation between the eastern and the western carrots was observed as expected. However, eastern accessions showed substantial levels of admixture (not observed in the Indian breeding lines), and the landraces from Turkey (E07) and Pakistan (E13) were attributed to the western cluster (Fig. 3a). At *K* = 3, the Indian lines were separated from the rest of the eastern accessions except E02 and E20 from Pakistan and E05 from India, which grouped together with the Indian lines (Fig. 3b). Western carrot accessions showed very little admixture, except Criolla (W10). PCoA results also reflected a more complex diversity structure within the eastern gene pool (Fig. 3c).

**Fig. 4:**
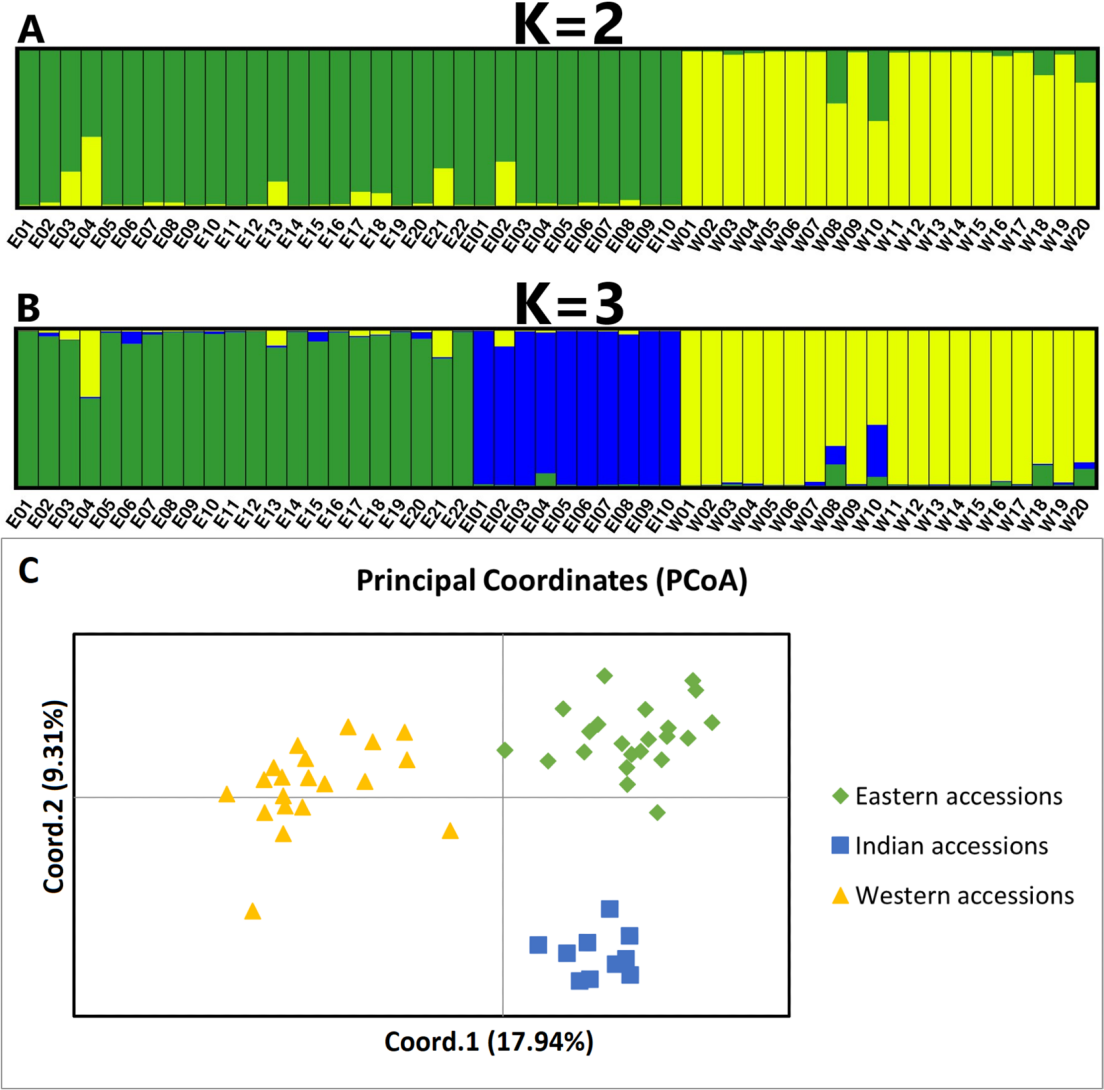
Genetic diversity structure in a collection of 52 carrot accessions representing eastern (E01-E22), Indian (EI01-EI10), and western (W01-W20) gene pools genotyped with 92 markers from the western *DcS*-ILP panel. STRUCTURE results at *K* = 2 (**a**) and *K* = 3 (**b**) and PCoA results (**c**) are shown

The population diversity structure inferred from genotyping with the western *DcS*-ILP panel, comprising 92 *DcSto* insertional polymorphisms (Supplementary Tables S5-S6), resulted in clear separation between the eastern and western carrot gene pools at *K* = 2 (Fig. 4a) and a division of the eastern gene pool into two clusters comprising the Indian breeding lines and the remaining eastern accessions at *K* = 3 (Fig. 4b), corroborated by PCoA (Fig. 4c).

### Performance of the eastern DcS-ILP panel

**Fig. 5:**
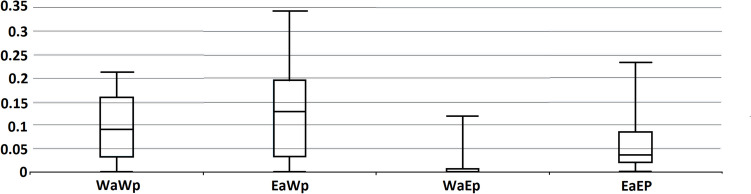
Box plots showing allelic frequencies with respect to the presence of *DcSto* copies (i.e., the “occupied” alleles), as revealed by *DcS*-ILP genotyping. WaWp, western accessions/western *DcS*-ILP panel; EaWp, eastern accessions/western *DcS*-ILP panel; WaEp, western accessions/eastern *DcS*-ILP panel; EaEp, eastern accessions/eastern *DcS*-ILP panel. *Y* axis shows frequency of the “occupied” allele

As described above, the newly developed eastern *DcS*-ILP genotyping panel produced more robust results with respect to the population structure of the eastern carrot gene pool. Despite the fact that both panels were able to separate the Indian breeding lines from the other eastern accessions, the eastern panel revealed the presence of genetic diversity structure, showing that accessions from Pakistan (E02, E20) and India (E05) were more closely related to the set of Indian breeding lines. Difference of the performance of markers based on reference vs. non-reference *DcSto* insertional polymorphisms was reflected by indices showing the performance of both panels (Table 2). While the western *DcS*-ILP panel identified very high level of polymorphism among the western accessions (%*P* = 97.83%), it was also effective in identifying polymorphisms in the eastern gene pool. In contrast, the newly developed eastern *DcS*-ILP panel identified few polymorphisms in the western gene pool (%*P* = 36.90%), while it was still highly effective in the eastern gene pool (Table 2). The reduced genotyping efficiency of the eastern panel in the western gene pool resulted from the fact that most *DcSto* non-reference insertions present in the eastern accessions were absent in the western accessions, i.e., they were all genotyped as “empty” homozygotes (Supplementary Table S3, Fig. 5).

**Table 2:** Indices of the performance of eastern and western *DcS*-ILP carrot genotyping panels

Indices*	Eastern *DcS*-ILP panel				Western *DcS*-ILP panel			
	Eastern accessions	Indian accessions	Western accessions	All accessions	Eastern accessions	Indian accessions	Western accessions	All accessions
Na	2.190	1.631	1.440	1.754	2.043	1.717	2.022	1.928
Ne	1.381	1.350	1.129	1.287	1.550	1.390	1.529	1.490
I	0.399	0.305	0.136	0.280	0.465	0.346	0.486	0.432
Ho	0.171	0.228	0.062	0.154	0.192	0.217	0.230	0.213
He	0.236	0.199	0.081	0.172	0.300	0.225	0.318	0.281
F	0.251	-0.110	0.220	0.138	0.335	0.061	0.272	0.239
%*P*	96.43%	55.95%	36.90%	63.10%	88.04%	66.30%	97.83%	84.06%

*Na, no. of alleles; *Ne*, no. of effective alleles; *I*, Shannon’s information index; *Ho*, observed heterozygosity; *He*, expected heterozygosity; *F*, fixation index; *%P*, percentage of polymorphic loci

## Discussion

Insertional polymorphisms resulting from the activity of TEs are a robust source of variability. They have been widely utilized to investigate genetic diversity in plants using a range of genotyping methodologies (Chang et al. 2001; Park et al. 2003; Casa et al. 2004; Lee et al. 2005). In carrot, a transposon display system detecting insertional polymorphisms of *DcMaster* elements, representing *PIF/Harbinger* superfamily, was developed (Grzebelus et al. 2007). Availability of plant genome assemblies allowed for more systematic annotation of TEs followed by the identification of insertional polymorphisms based on genome resequencing. *DcSto* MITE insertions were mined from the carrot genome (Iorizzo et al. 2016) and shown to be enriched in genic regions including introns and extremely polymorphic (Macko-Podgórni et al. 2019). It prompted us to develop a simple and reliable genotyping platform utilizing intronic *DcSto* polymorphisms identified via PCR with primers anchored in exons flanking the polymorphic insertion, named *DcS*-ILP (Stelmach et al. 2017). Such polymorphisms are generally biallelic (“occupied” vs. “empty” sites), but occasionally, additional variants may occur, resulting from insertions of unrelated TEs or other structural rearrangements. *DcS*-ILP markers provide a technically simple alternative to SNPs, allowing investigation on the fine genetic diversity structure, as shown by Stelmach et al. (2021).

Here, we report on an additional set of *DcS*-ILPs based on non-reference insertions identified in carrot accessions of Asian origin. Apart from being an additional source of polymorphisms, the application of eastern *DcS*-ILPs provided additional insight to the history of carrot improvement. In contrast to the reference insertions utilized by Stelmach et al. (2017), the insertions found in the eastern carrots occurred to be largely absent in the western gene pool (Fig. 5), which indicates a bottleneck related to the improvement step, i.e., transition from primitive eastern landraces to modern western cultivars. Apparently, only a fraction of insertions present in the eastern carrots was transferred to the western gene pool, resulting in the observed lower diversity of the latter. It has been long debated whether there was any domestication-related bottleneck in carrot, as early results did not provide such evidence (Iorizzo et al. 2013). A much more robust experiment involving resequencing of more than 600 accessions provided enough discriminatory power to confirm that there was a domestication bottleneck (Coe et al. 2023). Here, we argue that MITE insertional polymorphisms could provide a much more sensitive measure to detect selection-driven bottlenecks, also at the crop improvement stage. It seems that selection at this stage generally operated on standing variation, as the subset of *DcSto* insertions present in the western carrot likely originated from transposition events preceding the separation of both gene pools. Hence, while the eastern *DcS*-ILP panel occurred to be of limited use for the western carrots, it effectively identified genetic diversity in the eastern carrot gene pool, e.g., reflecting geographic origin of landraces. However, it is possible that a few insertions present at high frequencies only in the western accessions and absent in the genotyped eastern accessions, e.g., *DcS*-ILP608, *DcS*-ILP717, or *DcS*-ILP823 (Supplementary Tables S5-S6), represent insertions of *DcSto* copies which were active very recently in the improved gene pool. Macko-Podgórni et al. (2019) reported that subfamily *DcSto*7b is likely active in the cultivated carrot and suggested a related *Tc1/Mariner* autonomous element *DcMar*1 as a possible transposase donor. Alternatively, it could result from selection for the “occupied” allele at the improvement step, either simply because of its co-localization of the insertion with an advantageous gene variant or because of the actual functional effect of the insertion itself on the associated gene(s). Fagny et al. (2021) reported that MITEs can heavily influence the expression of adjacent genes resulting in altered phenotypes. In carrot, a MITE insertion was functionally associated with increased expression of a gene encoding a R2R3 MYB transcription factor resulting in the accumulation of anthocyanins in petioles (D’Amelia et al. 2023; Duan et al. 2023).

## Supplementary information

Below is the link to the electronic supplementary material.

Supplementary file1 (XLSX 108 KB)
